# Polarization‐Dependent Multiphoton‐Excited Self‐Trapped Emission in Alloyed 0D Rb_7_Bi_3_Cl_16_ Metal Halides via Sb^3+^ Doping

**DOI:** 10.1002/smsc.202500261

**Published:** 2025-06-21

**Authors:** Si Xiao, Haixia Zhu, Yao Liu, Zhaozhe Chen, Defeng Xu, Weichang Zhou, Zhihui Chen, Shan Liang, Hui Tong, Xueyi Guo, Jun He

**Affiliations:** ^1^ Hunan Key Laboratory of Nanophotonics and Devices School of physics Central South University 932 South Lushan Road Changsha Hunan 410083 China; ^2^ Department of Physics Hunan Normal University Changsha 410081 China; ^3^ School of Metallurgy and Environment Central South University Changsha 410083 China

**Keywords:** multiphoton photoluminescence, polarization‐dependent emission, self‐trapped excitons, soft lattice

## Abstract

Regulating the electronic structure by doping can promote photoluminescence emission of low‐dimensional metal halides for developing white‐light‐emitting devices. Here, 0D metal halides Rb_7_Bi_3_Cl_16_ have achieved a transition from nonluminescence to effective self‐trapped excitons (STEs) emission after Sb^3+^ ion doping at room temperature. The femtosecond transient absorption spectrum reveals the nonradiative recombination was suppressed, whose lifetimes change from 93.9 ps to 3.6 ns after doping Sb^3+^ ion. Moreover, the Rb_7_Bi_3_Cl_16_:Sb^3+^ exhibits polarization‐dependent two‐photon photoluminescence and three‐photon photoluminescence in the excitation wavelength range of 900–1200 nm, which further confirmed that STEs emission is extrinsic STEs by lattice deformation rather than defects. This work suggests that Sb^3+^ ion doping can effectively improve the luminescence properties of low‐dimensional metal halides and promote the application potential in high‐order nonlinear photoelectric field.

## Graphical abstract

The zero‐dimensional metal halides Rb_7_Bi_3_Cl_16_ can achieve a transition from non‐luminescence to effective polarization‐dependent multi‐photon STEs emission after Sb^3+^ ion doping. The mechanism of high‐efficiency STEs in doped Rb_7_Bi_3_Cl_16_ is the non‐radiative recombination was suppressed, whose lifetimes changed from 93.9 ps to 3.6 ns after doping Sb^3+^, revealed by femtosecond transient absorption spectrum.

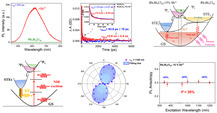

## 1 Introduction

Due to the potential application in the solid‐state lighting industry, the white‐light‐emitting (WLE) has attracted much attention in recent years.^[^ ^1^, ^2^, ^3^, ^4^ ^]^ The self‐trapped excitons (STEs) offer the opportunity for WLE devices developing by virtues of their broadband emission (BE), strongly Stokes‐shift, and long lifetimes.^[^ ^5^, ^6^, ^7^ ^]^ Metal halides can produce STEs emission due to their unique soft lattices.^[^ ^8^, ^9^, ^10^ ^]^ Especially, investigation has demonstrated that reducing the structural dimension of halides to 2D, 1D, or even 0D can promote the formations of STEs.^[^ ^11^, ^12^, ^13^ ^]^ But low dimension metal halides usually exhibit weak optical properties due to strong exciton‐phonon coupling or the indirect bandgap nature, which limits their application.^[^ ^14^, ^15^, ^16^, ^17^ ^]^

Fortunately, doping d‐, f‐, and s‐electron ions such as Mn^2+^, Cr^3+^, Tb^3+^, In^3+^, and Sb^3+^ into metal halides can effectively improve the luminescence efficiency,^[^ ^18^, ^19^, ^20^, ^21^ ^]^ which has become the research focus in recent years. In particular, In^3+^ and Sb^3+^ ions with ns^2^ outermost electron configuration can modulate metal halides electronic structures and boost photoluminescence (PL) emission in metal halides.^[^ ^22^, ^23^, ^24^ ^]^ For example, broadband STEs emission with a quantum yield (QY) of 75.89% was achieved in the alloyed Cs_2_NaInCl_6_: Sb^3+^ crystals by breaking the parity forbidden transition rule and modulating the density of state population.^[^ ^25^ ^]^ The QY of the 0D metal halide Rb_3_BiCl_6_ reached 33.6% after Sb^3+^ ion doping by suppressing the exciton‐phonon interactions.^[^ ^26^ ^]^ However, the exact electronic and structural nature of these STEs species remain incomplete understanding.^[^ ^27^, ^28^ ^]^ Sometimes, the phenomena of BE have been attributed simply to intrinsic STEs caused by photoinduced transient lattice distortion,^[^ ^29^, ^30^ ^]^ while several indicate that extrinsic STEs also can produce BE.^[^ ^31^, ^32^ ^]^ Besides, some believe that the BE originates from defects (vacancies or impurities) because the single crystals show diverse emission intensities under different preparation methods.^[^ ^33^, ^34^ ^]^

The multiphoton STEs emission and polarization response exhibited by some inorganic metal halides recently could be the key to resolve these disputes. The high‐order STEs emission were observed in Cs_2_TeCl_6_ crystal.^[^ ^35^ ^]^ Chen et al. found that Na^+^/Bi^3+^‐alloyed Cs_2_AgInCl_6_ can absorb six photons and realized multiphoton STEs emission.^[^ ^36^ ^]^ Furthermore, STEs emission originated from the lattice distortion, which may induce the deformation of the excited state lattice and lead to the anisotropy of the dipole moment, thus resulting in the anisotropy of PL.^[^ ^27^ ^]^ For example, Zhao et al. have observed that the inorganic metal halides Cs_2_KBiCl_6_ nanocrystals show the polarization‐dependent STEs emission.^[^ ^37^ ^]^ However, the enhanced emission efficiency of white light, multiphoton emission, and polarization properties promoted by doping Sb^3+^ ions at low‐dimensional metal halides are still rarely reported.

Based on the above considerations, we achieved a polarization‐dependent multi‐photon STEs emission in the 0D metal halides Rb_7_Bi_3_Cl_16_ by Sb^3+^ ion doping at room temperature (RT). The origin of high‐efficiency STEs in Rb_7_Bi_3_Cl_16_: Sb^3+^ was revealed by femtosecond transient absorption (TA) spectrum. The nonradiative recombination is slowed obviously in Rb_7_Bi_3_Cl_16_: Sb^3+^ that is associated with the reduction of octahedral distortion caused by electron–phonon interaction. Meanwhile, the 0D metal halides Rb_7_Bi_3_Cl_16_: Sb^3+^ possess the multiphoton polarization‐dependent STEs emission properties, which further demonstrated that the STEs emission is extrinsic STEs rather than defects. This work provides an in‐depth study of the origin of STEs emission enhancement in low‐dimensional alloy metal halides, paving the way for developing WLE materials for multifunctional applications via doping engineering.

## 2 Results and Discussion

**Figure 1:**
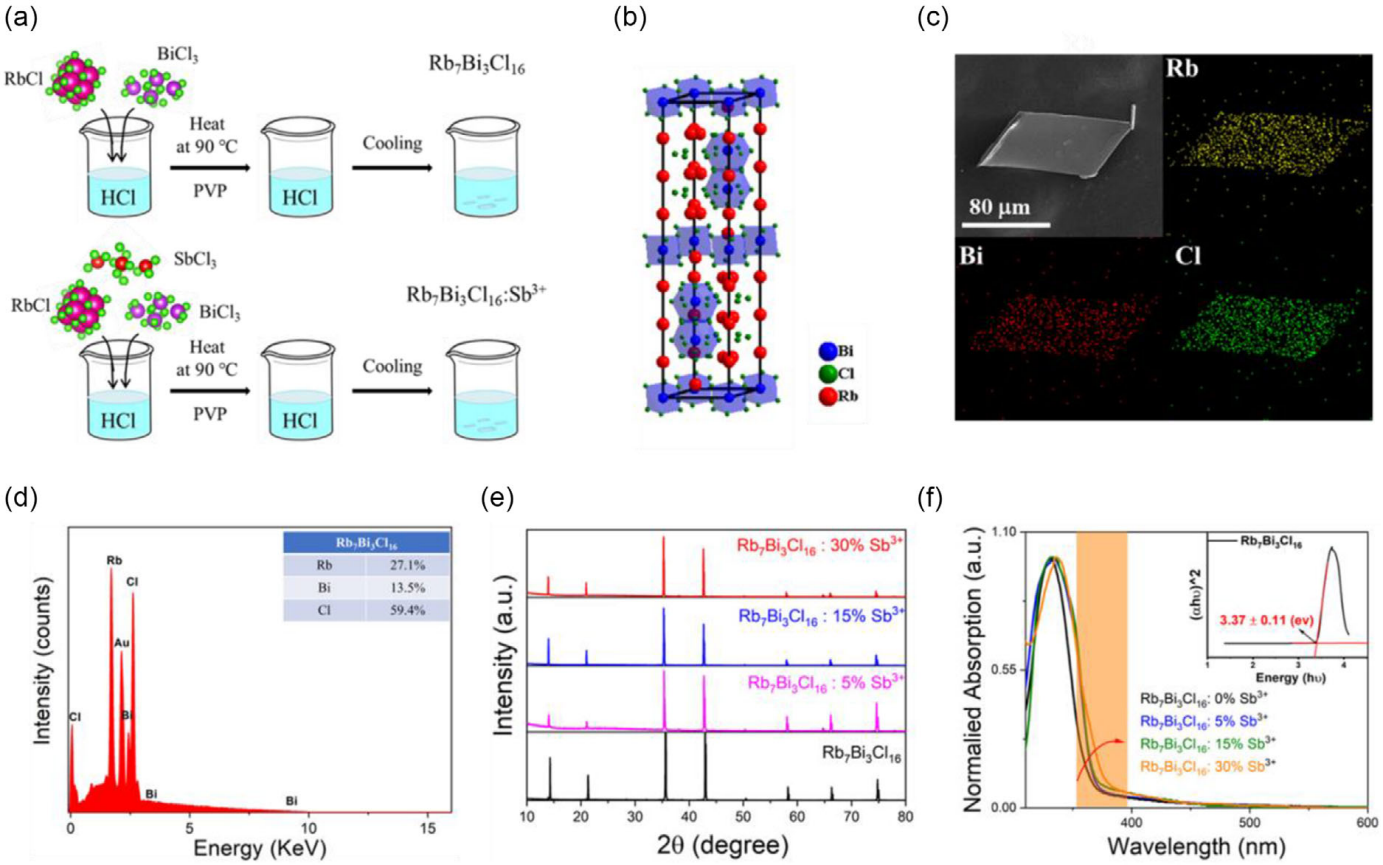
Structure characterization of synthesized Rb_7_Bi_3_Cl_16_: Sb^3+^. a) Schematic procedures for the synthesis of Rb_7_Bi_3_Cl_16_ (top) and Rb_7_Bi_3_Cl_16_: Sb^3+^ (bottom). b) The crystal structure of Rb_7_Bi_3_Cl_16_. c) SEM image and elemental mappings of Rb_7_Bi_3_Cl_16_ nanosheets. d) EDS spectrum of Rb_7_Bi_3_Cl_16_ crystals. e) SCXRD patterns of Rb_7_Bi_3_Cl_16_ and Rb_7_Bi_3_Cl_16_: x% Sb^3+^ (*x* = 5, 15, and 30). f) Normalized absorption spectra of the as‐prepared Rb_7_Bi_3_Cl_16_ and Rb_7_Bi_3_Cl_16_: x% Sb^3+^ (*x* = 5, 15, and 30). The inset shows Tauc plot of Rb_7_Bi_3_Cl_16_.

Rb_7_Bi_3_Cl_16_ single crystals were synthesized by hydrothermal method, as described in the **Figure** **1**a (top). The doping concentration of Rb_7_Bi_3_Cl_16_: Sb^3+^ can be adjusted by changing the molar ratio of Sb^3+^ to Bi^3+^ in the precursor solution (bottom). The Rb_7_Bi_3_Cl_16_ belongs to the trigonal P$\bar{3}$1C (163) space group.^[^ ^38^ ^]^ As shown in Figure 1b,^[^ ^39^ ^]^ the Rb_7_Bi_3_Cl_16_ consists of two asymmetric Bi atoms, five Rb atoms, and seven Cl atoms, while each Bi atom is coordinated with six adjacent Cl atoms forming discrete [BiCl_6_]^3−^ octahedra or [Bi_2_Cl_6_]^4−^ edge‐sharing dimers. Compared with that of the terminal chlorine bonds, the Bi‐Cl bonds for [Bi_2_Cl_6_]^4−^ edge‐sharing dimers are longer, which indicate that [Bi_2_Cl_6_]^4−^ edge‐sharing dimers is prone to distortion. Such distortion is associated with photophysical processes, which usually leads to broadband STEs emissions in metal halide.^[^ ^2^, ^5^ ^]^ The Figure 1c shows the scanning electron microscopy (SEM) and corresponding element mapping of Rb_7_Bi_3_Cl_16_. The Rb, Bi, and Cl elements are homogeneously distributed over whole regular Rb_7_Bi_3_Cl_16_ nanosheets. The energy‐dispersive X‐ray spectroscopy (EDS) result for the as‐prepared Rb_7_Bi_3_Cl_16_ crystal was showed in Figure 1d that displays peaks corresponding to Rb, Bi, and Cl, while the ratios are 7:3:16.

The stoichiometric ratio of Rb_7_Bi_3_Cl_16_: *x*% Sb^3+^ (*x* = 5, 15, and 30) is further measured by element compositions obtained from EDS analysis, as displayed in the Figure S1–S3, Supporting Information, which manifested that Sb^3+^ ions were doped into Rb_7_Bi_3_Cl_16_ crystal. The single crystal X‐ray diffraction (SCXRD) measurement of Rb_7_Bi_3_Cl_16_: *x*% Sb^3+^ (*x* = 5, 15, and 30) is shown in Figure 1e. The diffraction peaks of Rb_7_Bi_3_Cl_16_: *x*% Sb^3+^ (*x* = 5, 15, 30) do not shift with increasing Sb^3+^ ion concentration, indicating that the crystal structure of Rb_7_Bi_3_Cl_16_: *x*% Sb ^3+^ (*x* = 5, 15, and 30) is consistent with Rb_7_Bi_3_Cl_16_ crystal, which is consistent with previous reports.^[^ ^40^ ^]^

As shown in Figure 1f, the Rb_7_Bi_3_Cl_16_ and Rb_7_Bi_3_Cl_16_: *x*% Sb^3+^ (*x* = 5, 15, and 30) show similar absorption character, with absorption peak at 330 nm. The optical bandgap of Rb_7_Bi_3_Cl_16_ calculated is 3.37 ± 0.11 eV by Tauc method (Figure 1f inset), which is consistent with the value reported.^[^ ^40^ ^]^ However, the absorption of Rb_7_Bi_3_Cl_16_: x% Sb^3+^ (*x* = 5, 15, and 30) gradually shifts to longer wavelength and enhances the absorption between 400 and 500 nm with increasing Sb^3+^ ion, which is consistent with general rule with Sb^3+^ ion doping.^[^ ^26^, ^41^ ^]^

PL performance of Rb_7_Bi_3_Cl_16_: x% Sb^3+^ (*x* = 5, 15, and 30) has been displayed in Figure S4–S6, Supporting Information. Considering that Rb_7_Bi_3_Cl_16_: 15% Sb^3+^ has the highest quantum yield among them, it was chosen as a showcase of example for investigation. In these experiments, the sample damage by the laser illumination were no observed.

**Figure 2:**
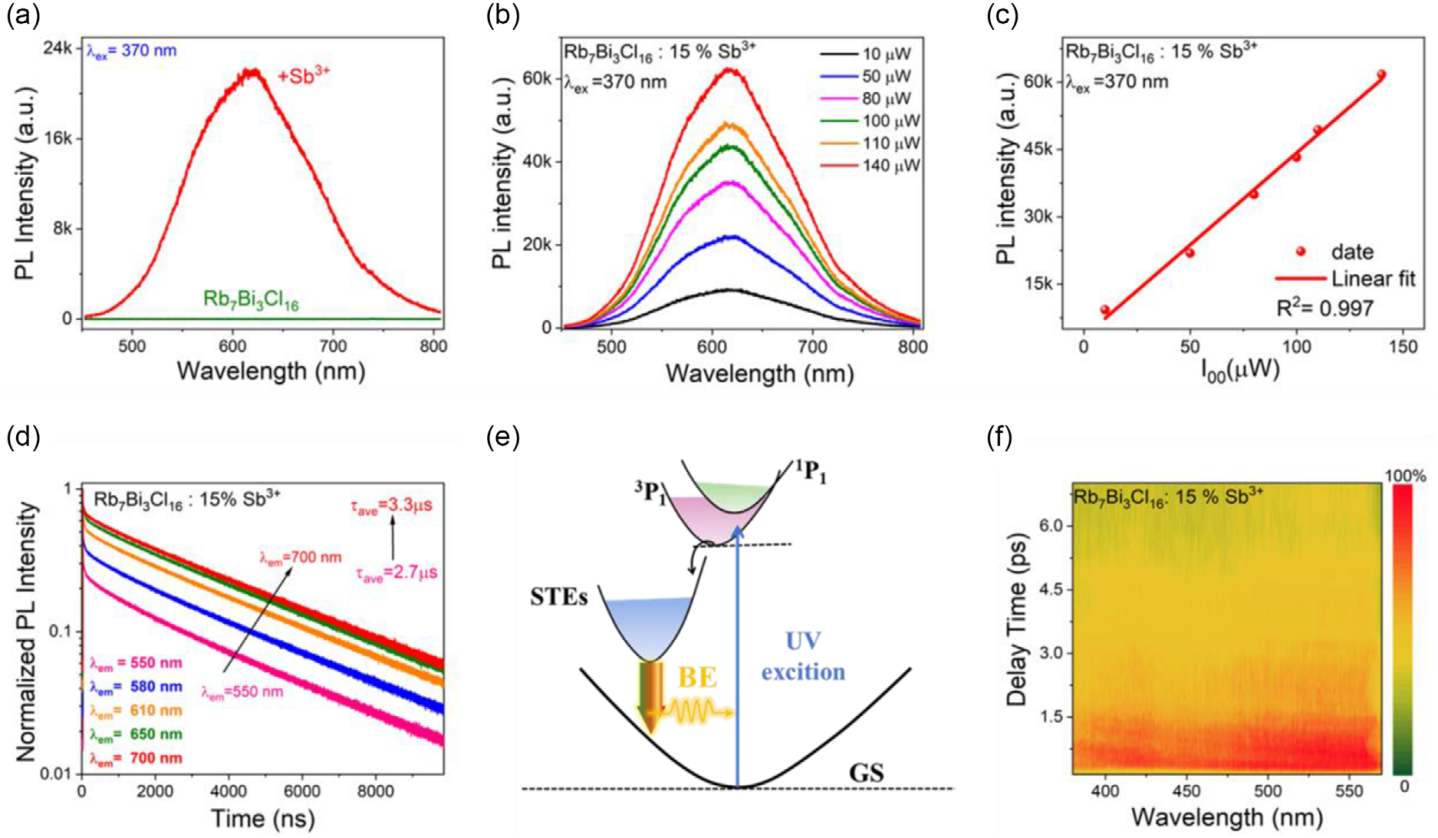
PL performance of Rb_7_Bi_3_Cl_16_: Sb^3+^ a) PL spectra of Rb_7_Bi_3_Cl_16_ and Rb_7_Bi_3_Cl_16_: Sb^3+^. b) The power‐dependent PL spectra of the Rb_7_Bi_3_Cl_16_: 15% Sb^3+^ crystals under 370 nm excitation. c) PL intensity of Rb_7_Bi_3_Cl_16_: 15% Sb^3+^ as a function of excitation power. d) PL decay curves of Rb_7_Bi_3_Cl_16_: 15% Sb^3+^ monitored at different wavelengths under 370 nm excitation. e) Illustration of the electron transition process involved in STEs emission. f) Contour plots of the *fs*‐TA spectra of Rb_7_Bi_3_Cl_16_: 15% Sb^3+^ as a function of wavelength and delay time.

As shown in **Figure** **2**a, the Rb_7_Bi_3_Cl_16_: Sb^3+^ achieved a transition from non‐luminescence to effective PL emission, showing a BE spanning from 450 to 800 nm with a full width at half maxima (FWHW) of 120 nm. As demonstrated in Figure S7, Supporting Information, the PL excitation (PLE) spectrum (emission wavelength at 619 nm) of Rb_7_Bi_3_Cl_16_: 15% Sb^3+^ shows two excitation peaks at 367 and 310 nm, respectively, which can be attributed to the ^1^S_0_ → ^1^P_1_, and ^1^S_0_ → ^3^P_1_ transition, respectively.^[^ ^40^ ^]^ Under 370 nm excitation, Rb_7_Bi_3_Cl_16_: 15% Sb^3+^ shows an emission band at 619 nm with a large stokes shift of 252 nm, which can be attributed to the transition of ^3^P_1_ → ^1^S_0_. The power‐dependent PL measurements (10–140 μw) of Rb_7_Bi_3_Cl_16_: 15% Sb^3+^ (Figure 2b) were measured. As shown in Figure 2c, the clear linear behavior (slope = 0.98, *R* ^2^ = 0.997) suggests the low possibility of defect‐assisted emission processes, because permanent defects may saturate at high excitation intensities.^[^ ^42^ ^]^ In addition, average fitting lifetime of Rb_7_Bi_3_Cl_16_: 15% Sb^3+^, which increases gradually from 2.7 to 3.3 μs, was also observed when the emission wavelength changed from 550 to 700 nm (Figure 2d). This trend likely arises because excited carriers can transfer from shallower to deeper trapped states, resulting in a longer lifetime at longer wavelengths.^[^ ^43^ ^]^

Considering the BE, long lifetime, and a large Stokes shift of 252 nm (Figure S7, Supporting Information) displayed, the emission of Rb_7_Bi_3_Cl_16_: 15% Sb^3+^should belong to STEs emission. The emission diagram of the STEs is shown in Figure 2e. Under 370 nm excitation, the triplet ^3^P_1_ electronic state undergoes ^3^P_1_ → ^1^S_0_ radiative transitions, forming the broadband STEs emission process. The PL spectrum of Rb_7_Bi_3_Cl_16_: 15% Sb^3+^ from low excitation energy (3.35 eV and 370 nm) to high excitation energy (4.51 eV and 275 nm) was also measured and found that they shift from low to high energy correspondingly (Figure S8, Supporting Information). Moreover, the Rb_7_Bi_3_Cl_16_ shows a relatively narrow PL peak at 430 nm (Figure S9, Supporting Information), which further corroborates the existence of STEs state with a deep self‐trapping depth in Rb_7_Bi_3_Cl_16_: Sb^3+^. Combined with Figure 2d, the slight blue shift of:Rb_7_Bi_3_Cl_16_: Sb^3+^ under high energy excitation may be due to the emission of electrons transferred from the ^3^P_1_ state to different self‐trapped states. This phenomenon has been observed in the Rb_3_BiCl_6_ system.^[^ ^26^ ^]^ Further, the positive photoinduced absorption (PIA) of the pseudo color TA spectra plot could be considered a direct evidence for STEs. In Figure 2f, the positive PIA of the pseudo color TA spectra plot in Rb_7_Bi_3_Cl_16_: 15% Sb^3+^ was observed, in the probe region from 380 to 580 nm with 325 nm laser excitation.

**Figure 3:**
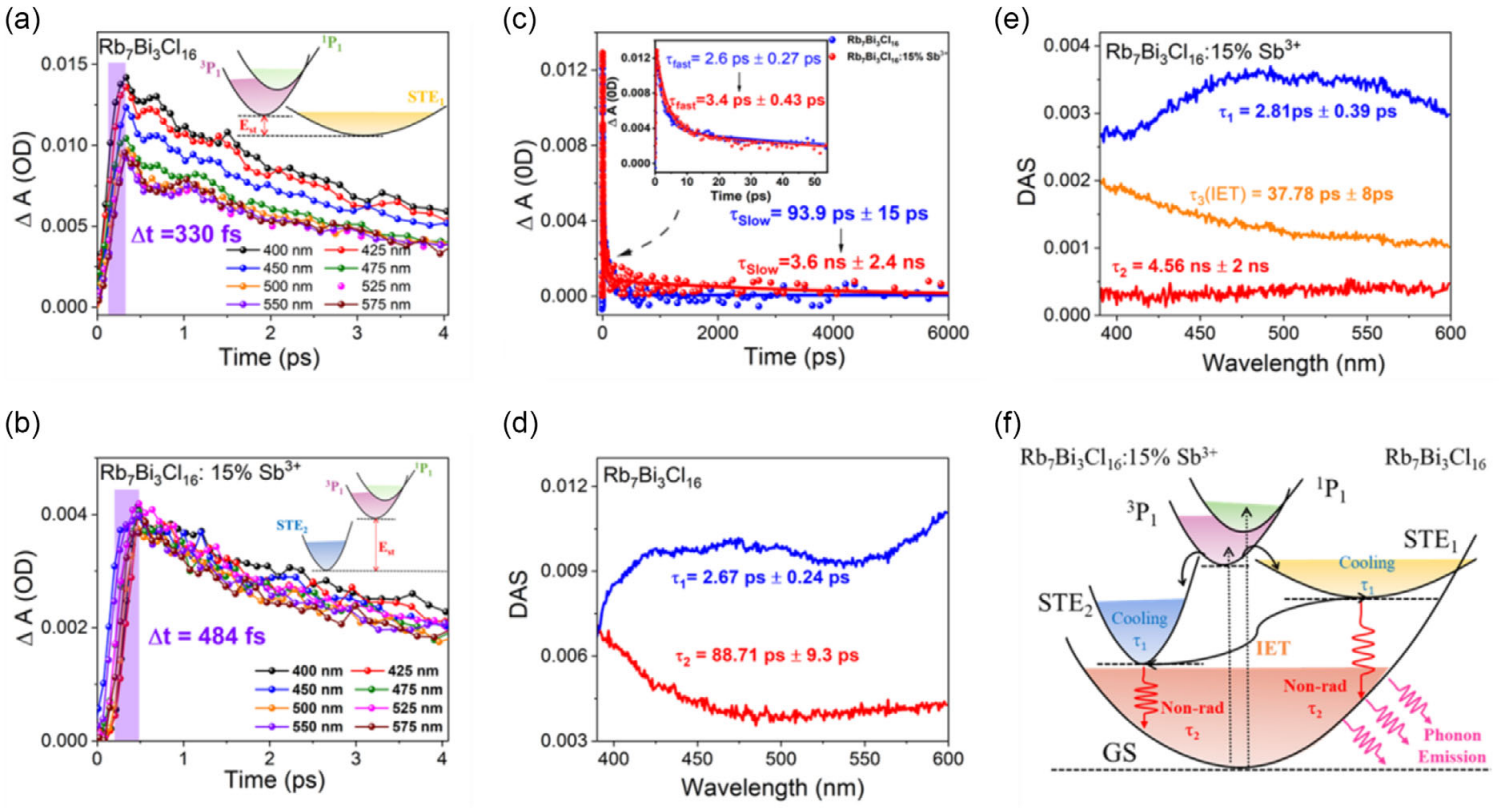
Dynamics characterization of Rb_7_Bi_3_Cl_16_: Sb^3+^ crystal with 325 nm laser excitation. a) PIA onsets of Rb_7_Bi_3_Cl_16_ probed at different probe wavelengths. The inset shows energy‐level distortion of Rb_7_Bi_3_Cl_16_. b) PIA onsets of Rb_7_Bi_3_Cl_16_:15% Sb^3+^ probed at different wavelengths. The inset shows energy‐level distortion of Rb_7_Bi_3_Cl_16_:15% Sb^3+^. c) PIA decay curves of Rb_7_Bi_3_Cl_16_ and Rb_7_Bi_3_Cl_16_:15% Sb^3+^ probed at 535 nm. The inset shows the decay process at beginning stage. d) The kinetics of Rb_7_Bi_3_Cl_16_ obtained by global fitting algorithm. e) The kinetics of Rb_7_Bi_3_Cl_16_:15% Sb^3+^ obtained by global fitting algorithm. f) Schematic diagram of the recombination process of Rb_7_Bi_3_Cl_16_ and Rb_7_Bi_3_Cl_16_:15% Sb^3+^. IET: interband energy transfer.

The STEs formation process and nonradiative decay in low‐dimensional perovskites could be quantitatively measured through the femtosecond TA (*fs*‐TA) spectrum. In the experiments, the thermal effect can be negligible, because fs laser pulses are less than the heat exchange time between the electrons and the lattice. As shown in **Figure** **3**a,b, the rise time of PIA from 400 to 575 nm is fitted as 330 fs and 484 fs for Rb_7_Bi_3_Cl_16_ and Rb_7_Bi_3_Cl_16_: 15% Sb^3+^, respectively. These ultrafast rise times are very close to the STEs formation time (≈212 fs) of Rb_3_BiCl_6_ previously reported by Kuang et al. in 2021,^[^ ^26^ ^]^ so these dynamics processes are also related to Jahn–Teller distortion. However, compared with Rb_7_Bi_3_Cl_16_: 15% Sb^3+^ crystal, the faster STEs formation process as 330 fs in Rb_7_Bi_3_Cl_16_ crystal observed, may originate from the extreme exciton‐phonon coupling in Rb_7_Bi_3_Cl_16_. It demonstrates a smaller energy barrier between the FE state and the STEs state (inset of Figure 3a), which is easier to transfer.^[^ ^44^ ^]^ In addition, the deep STEs state in Rb_7_Bi_3_Cl_16_: 15% Sb^3+^ could be further confirmed by Raman spectroscopy. As shown in Figure S10a, the intensity of the Raman peak of Rb_7_Bi_3_Cl_16_: 15% Sb^3+^ is significantly lower than that of Rb_7_Bi_3_Cl_16_, which indicates that the vibrational mode of the Rb_7_Bi_3_Cl_16_: 15% Sb^3+^ has substantially weaker coupling strength. It may cause the reduced vibrational overlap between the excited and ground state vibrational wave functions, that is, the smaller lattice deformation, as shown in Figure S10b, Supporting Information, indicating the deep STEs state in Rb_7_Bi_3_Cl_16_: 15% Sb^3+^ (inset of Figure 3b).

The carrier decay curve can directly show the process of non‐radiative recombination being suppressed in Rb_7_Bi_3_Cl_16_: 15% Sb^3+^. The Rb_7_Bi_3_Cl_16_ and Rb_7_Bi_3_Cl_16_: 15% Sb^3+^ exhibit the same fast relaxation process (inset of Figure 3c). But Rb_7_Bi_3_Cl_16_: 15% Sb^3+^ exhibits a slower relaxation process (Figure 3c), compared with Rb_7_Bi_3_Cl_16_, which proves that Sb^3+^ ion doped into Rb_7_Bi_3_Cl_16_ can effectively suppress the nonradiative recombination process, promoting luminescence enhancement.

In order to clarify the carrier relaxation process of Rb_7_Bi_3_Cl_16_ and Rb_7_Bi_3_Cl_16_: 15% Sb^3+^, the global fitting was used to fit data. As shown in Figure 3d, the Rb_7_Bi_3_Cl_16_ of decay consists of two processes: *τ*_1_ = 2.67 ps ± 0.24 ps and *τ*_2_ = 88.71 ps ± 9.30 ps, while the decay of Rb_7_Bi_3_Cl_16_: 15% Sb^3+^ consists of three processes: *τ*_1_ = 2.81ps ± 0.39 ps, *τ*_2_ = 4.56 ns ± 2.00 ns, and *τ*_3_ = 37.78 ps ± 8.00 ps. The *τ*_1_ of these two materials both originated from the cooling of hot STEs.^[^ ^26^ ^]^ The *τ*_2_ can be assigned to the nonradiative recombination of STEs.^[^ ^26^ ^]^ The nonradiative recombination time of Rb_7_Bi_3_Cl_16_ (*τ*_2_ = 88.71 ps ± 9.30 ps) is much faster than that of Rb_7_Bi_3_Cl_16_: 15% Sb^3+^ (*τ*_2_ = 4.56 ns ± 2.00 ns), which leads to its fast relaxation to ground state via nonradiative recombination rather than luminescence, resulting in the PL of Rb_7_Bi_3_Cl_16_ being extremely quenched at RT. The additional middle lifetime (*τ*_3_ = 37.78 ps ± 8. 00 ps) for Rb_7_Bi_3_Cl_16_: 15% Sb^3+^ can be attributed to the energy transfer from STE_1_ to STE_2_ states. Combining all the above analyses, the overall kinetic analysis is summarized in Figure 3f.

**Figure 4:**
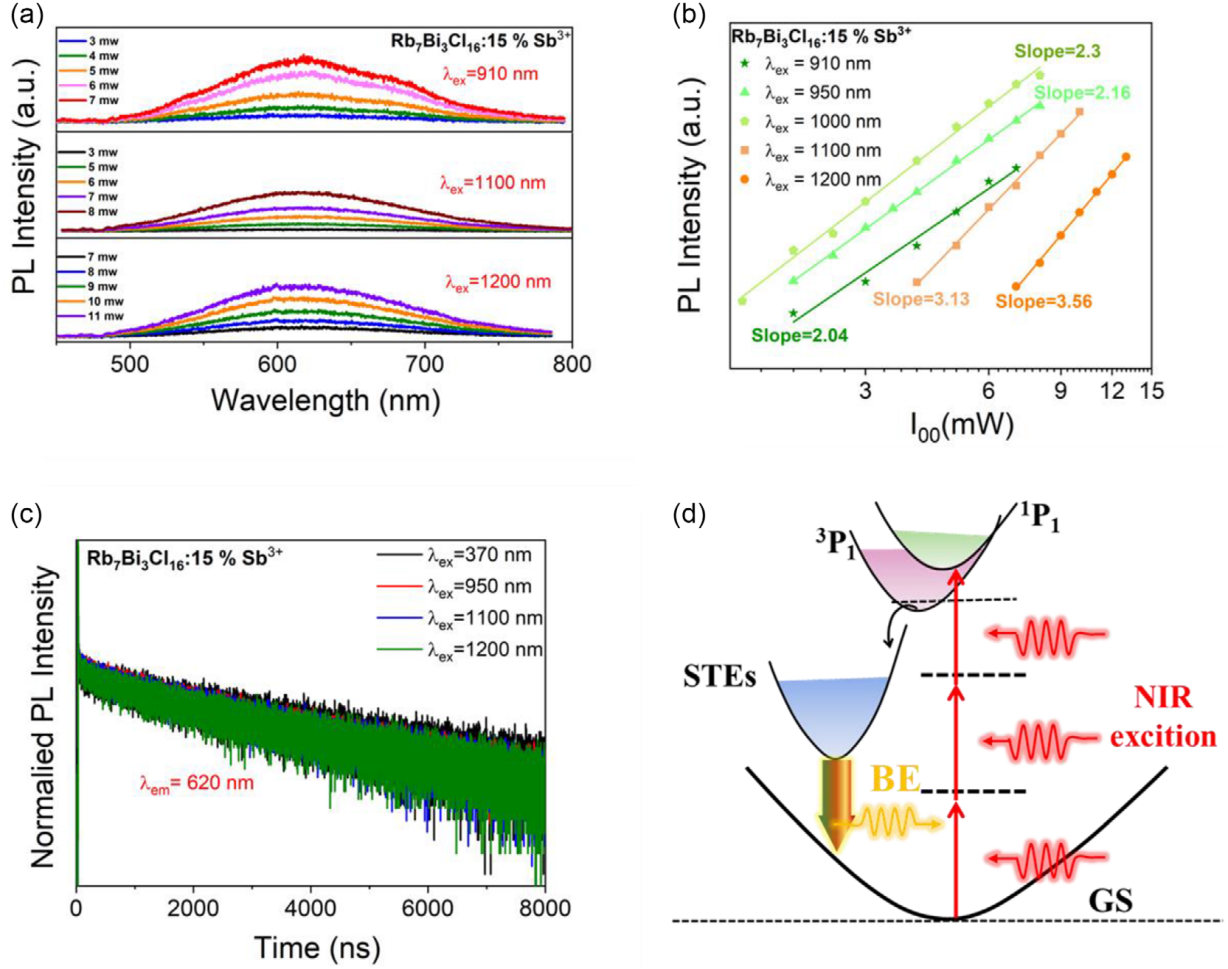
Multiphoton‐excited SETs emission characterization of Rb_7_Bi_3_Cl_16_: Sb^3+^ crystal. a) Multiphoton‐excited PL spectra of the Rb_7_Bi_3_Cl_16_: 15% Sb^3+^ with different excitation wavelengths at 910, 1100, and 1200 nm, respectively. b) The emission intensity as a function of excitation power under different excitation wavelengths. c) Single‐and multiphoton‐excited STEs decay curves at 620 nm emission for Rb_7_Bi_3_Cl_16_: 15% Sb^3+^ crystals. d) Schematic of multiphoton‐excited STEs emission process for Rb_7_Bi_3_Cl_16_: Sb^3+^ crystals.

Multiphoton‐excited SETs emission is possible, benefiting from the significant suppression of non‐radiative recombination after the doping of Sb^3+^ ion. As shown in **Figure** **4**a, the Rb_7_Bi_3_Cl_16_:15% Sb^3+^ exhibits the STEs emission with different excitation wavelengths at 910, 1100 and 1200 nm, respectively. It is also observed that the emission intensity increases with increasing thickness. The dependence of the PL intensity (*I*) on excitation power (*L*) is given by the $I\approx L^{a}$ power law dependence,^[^ ^35^ ^]^ the *n*‐photon absorption represented by the exponent α. The slopes of SETs emission intensities versus powers at different near‐infrared excitation wavelengths were shown in Figure 4b. The slope values of 2.04, 2.16, 2.30, 3.13, and 3.56 indicate that the two‐photon PL (2PPL) and three‐photon PL (3PPL) processes occur in Rb_7_Bi_3_Cl_16_:15% Sb^3+^. The slope of 1200 nm excitation deviates from 3 so much, indicating the existence of higher‐order absorption processes under high light intensity. Figure S11, Supporting Information, shows the normalized 1PPL, 2PPL, and 3PPL spectrum of the Rb_7_Bi_3_Cl_16_:15% Sb^3+^. The multiphoton‐excited emission spectra and the 1PPL spectra are indistinguishable, which suggests that the same emission states are involved in the cases of single‐photon and multiphoton excitation. Furthermore, the decay curves of time‐resolved PL (TRPL) do not change between single‐photon and multiphoton excitation of Rb_7_Bi_3_Cl_16_:15% Sb^3+^, which clearly indicate the same STEs radiation transition for the linear and multiphoton‐excited processes. As illustrated in Figure 4d, upon near‐infrared (NIR) excitation, the Rb_7_Bi_3_Cl_16_: Sb^3+^ absorb two or three photons from the NIR pulses to reach the triplet ^3^P_1_, and then the triplet ^3^P_1_ electronic state undergoes ^3^P_1_ → ^1^S_0_ radiative transitions, forming the broadband STEs emission process. Considering a deeper penetration depth for near‐infrared laser excitation, it is speculated that this BE peak originates from STEs that appear inside the material, which further exclude the possibility of emission caused by impurity defect states.^[^ ^45^ ^]^

**Figure 5:**
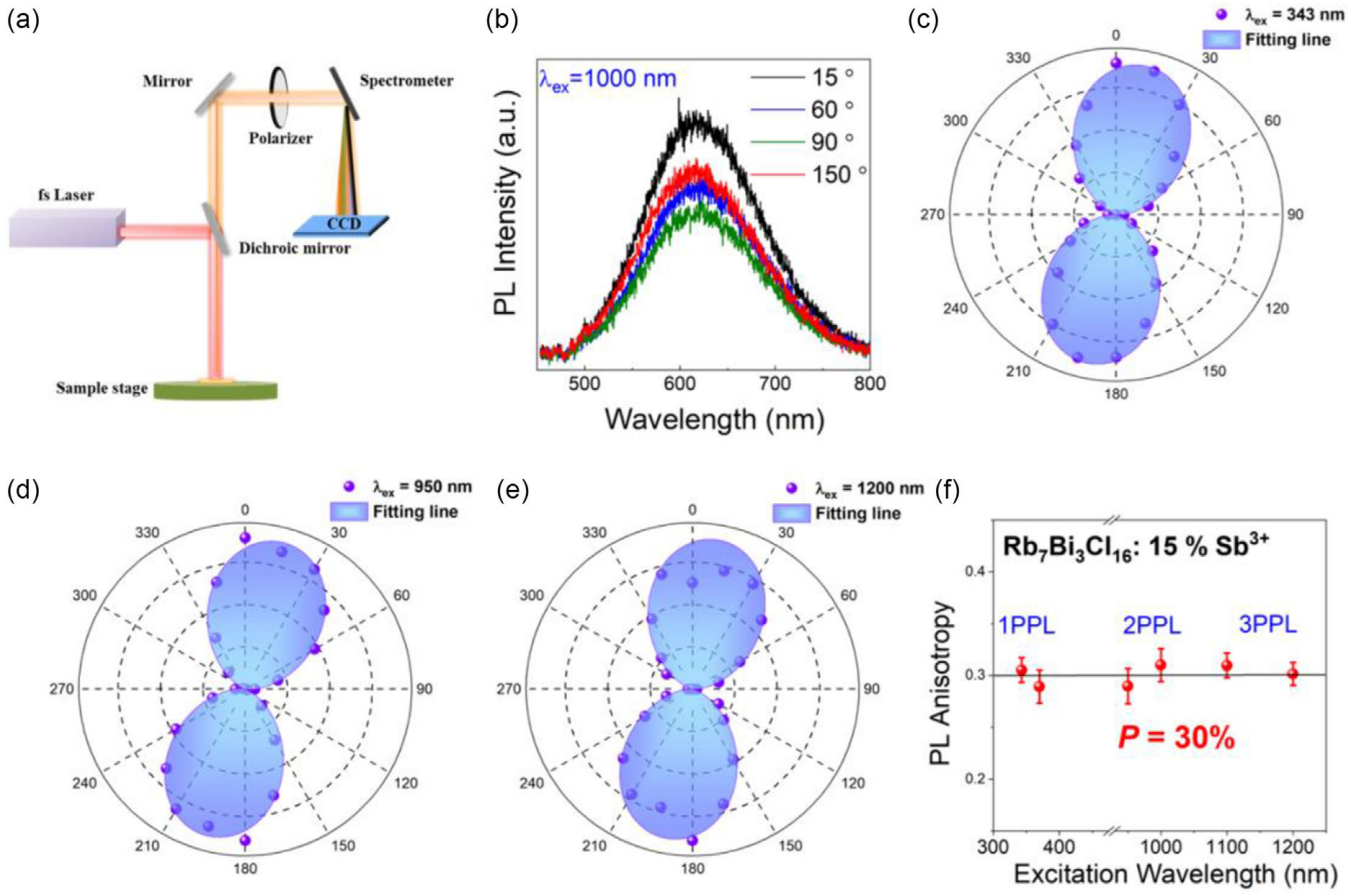
Anisotropy multiphoton‐excited SETs emission characterization of Rb_7_Bi_3_Cl_16_: 15% Sb^3+^ crystal. a) Schematic diagram of the device for measuring emission anisotropy properties. b) Emission anisotropy spectrum of Rb_7_Bi_3_Cl_16_: 15% Sb^3+^ crystal at different linear polarizer angle under 1100 nm excitation wavelength. c–e) Polar plot of integrated PL intensity of Rb_7_Bi_3_Cl_16_: 15% Sb^3+^ as a function of *θ* under 343, 950, and 1200 nm excitation wavelengths, respectively. f) STEs emission *P* values of Rb_7_Bi_3_Cl_16_: 15% Sb^3+^ at different excitation wavelengths.

Surface defects or impurities are usually randomly distributed, which lead to isotropic emission of PL.^[^ ^27^, ^46^, ^47^ ^]^ However, STEs emission of Rb_7_Bi_3_Cl_16_: Sb^3+^ involves lattice distortion, which may cause a deviation between the emission transition dipole moment and the excitation transition dipole moment, resulting in anisotropic PL emission.^[^ ^37^ ^]^ Therefore, the polarized multiphoton‐excited PL of Rb_7_Bi_3_Cl_16_: Sb^3+^ was carried out. During the measurement, excitation laser was focused onto the single crystals located on quartz. The polarization of the excitation light was kept unchanged, while a linear polarizer is inserted in the detection channel to collect the PL spectra from 0° to 360°, as illustrated in **Figure** **5**a. PL spectra of Rb_7_Bi_3_Cl_16_:15% Sb^3+^ crystal under 1000 nm excitation were shown in Figure 5b, which were obtained by varying linear polarizer angle of detection channel and collecting the whole PL signal. The intensity of the PL peak clearly varies with the angle of the polarizer. Figure 5c–e exhibits a polar plot of integrated PL intensity as a function of *θ* under 343, 950, and 1200 nm excitation wavelengths, respectively. The polarization dependent 1PPL, 2PPL, and 3PPL displayed 2‐petal patterns, suggesting that Rb_7_Bi_3_Cl_16_: Sb^3+^ has the anisotropic STEs emissions properties, which has never been reported before. Particularly, the polarization‐dependent emission and absorption show almost the same phase (Figure S12, Supporting Information), which indicated that the polarized PL behavior of Rb_7_Bi_3_Cl_16_: Sb^3+^ may originate from the anisotropy of the transition dipole moment.^[^ ^48^ ^]^ The integrated PL intensity versus polarization angle can be well fitted by cos^2^ *θ* function, as shown in Figure 5c–e. Moreover, the degree of polarization (*P*) can be quantified by fitting the following equation^[^ ^27^ ^]^

(1) $$P=\frac{I_{\max}-I_{\min}}{I_{\max}+I_{\min}}$$

where $I_{\max}$ and $I_{\min}$ represent maximum and minimum PL peak intensity, respectively. By calculating the experimental data, Figure 5f shows *P* of Rb_7_Bi_3_Cl_16_:15% Sb^3+^ under different excitation wavelength. The *P* value of Rb_7_Bi_3_Cl_16_:15% Sb^3+^ is 0.305 and 0.289 for 343 and 370 nm excitation in 1PPL, respectively. The *P* of Rb_7_Bi_3_Cl_16_:15% Sb^3+^ is 0.289 and 0.310 for 950 and 1000 nm excitation in 2PPL, respectively. The *P* of Rb_7_Bi_3_Cl_16_:15% Sb^3+^ is 0.309 and 0.301 for 1100 and 1200 nm excitation in 3PPL, respectively. The degree of polarization is consistent at 0.3 in 1PPL, 2PPL, and 3PPL after excluding deviations, which suggests potential application of low‐dimensional metal halides in the field of polarized STEs, such as biological imaging and display lighting.

## 3 Conclusion

In summary, the Sb^3+^ ion‐doped 0D metal halides Rb_7_Bi_3_Cl_16_ with extrinsic STEs emission has been synthesized. The substitution of Bi^3+^ ion by a small amount of Sb^3+^ ion leads to a significant transition from nonluminescence to efficient STEs emission of Rb_7_Bi_3_Cl_16_ at RT. Femtosecond TA spectrum revealed that the effective STEs emission enhancement was ascribed to the slowed nonradiative recombination rate, as a results of exciton‐phonon interaction reduction at distorted octahedra by Sb^3+^ ion doping. Moreover, the Rb_7_Bi_3_Cl_16_:Sb^3+^ exhibits polarization‐dependent 2PPL and 3PPL under a wavelength range of 900–1200 nm. This work revealed mechanism of high‐performance luminescence of Rb_7_Bi_3_Cl_16_:Sb^3+^ metal halides, which may provide a guidance for the development of efficient STEs emission materials.

## 4 Experimental Section

4.1

4.1.1

##### Fabrication of 0D Metal Halides Rb_*7*_ *Bi*_*3*_ *Cl*_*16*_ *: Sb* ^3+^

A mixture of 117.8 g RbCl and 141.3 g BiCl_3_ was dissolved in 1.79 mL HCl solution. The 18 mg PVP was added to the precursor solution and was heated at 90 °C for 1 h and then slowly cooled to RT to get colorless Rb_7_Bi_3_Cl_16_ crystals. The Rb_7_Bi_3_Cl_16_: *x*% Sb^3+^ (*x* = 5, 15, and 30) were synthesized under the same condition except for adding a certain amount of SbCl_3_.

##### Characterizations Measurement

The morphology of Rb_7_Bi_3_Cl_16_: Sb^3+^ was characterized by the scanning electron microscope (SEM) (MIRA3 LMH). The elemental distributions are characterized by EDS equipment. The SCXRD was performed to analyse the phase. The UV‐visible absorption spectra of the Rb_7_Bi_3_Cl_16_: Sb^3+^ were obtained from the Agilent Cary 60 spectrophotometer.

##### Spectroscopy Measurement

An optical parametric amplifier (TOPAS, Orpheus) was utilized as the femtosecond laser source (1 MHz, 280 fs), which is generated by the mode‐locked Ti:sapphire laser (light conversion). The PL signals were measured in reflection geometry using inverted microscope (Nikon Eclipse Ti). Emission from the Rb_7_Bi_3_Cl_16_: *x*% Sb^3+^ (*x *= 5, 15, and 30) was collected with the 10× objective lens (N.A. = 0.30) and routed via a bundled optical fiber to a spectrometer (Acton, Spectra Pro 23ooi) coupled with CCD (Princeton Instruments). Time‐resolved PL (TRPL) was recorded on Pico Quant with the time‐correlated single‐photon counting (TCSPC) mode. RT Raman Spectroscopy was measured by a confocal In Via Qontor spectrometer with excitation wavelength at 532 nm. The PLQY was conducted using the Edinburgh FLS 1000 instrument with an excitation wavelength of 370 nm.

##### Femtosecond TA Spectroscopy Measurements

The ultrafast *fs*‐TA measurement was performed by the mode‐locked Ti:sapphire regenerative amplifier laser source (1 kHz, 35 fs) and the commercial Helions (Ultrafast systems) system. The pump wavelength is 325 nm, and the probe wavelength is a broadband probe super‐continuum (350–650 nm).

## Conflict of Interest

The authors declare no conflict of interest.

## Supporting information

Supplementary Material

## Data Availability

The data that support the findings of this study are available from the corresponding author upon reasonable request.
