# Supplementary material for: Polarization‐Dependent Multiphoton‐Excited Self‐Trapped Emission in Alloyed 0D Rb7Bi3Cl16 Metal Halides via Sb3+ Doping
Source: Small Sci. 2025 Jun 21;5(9):2500261. doi: 10.1002/smsc.202500261 (PMC12412464; doi:10.1002/smsc.202500261)
Supplement: Supplementary file 1 — Supplementary Material [file SMSC-5-2500261-s001.pdf]

Supporting Information

Title: Polarization-dependent Multi-Photon Excited Self-Trapped Emission in Alloyed Zero-dimensional $Rb_7Bi_3Cl_{16}$ Metal halides Via Sb^{3+} Doping

Author(s), and Corresponding Author(s)*: **Si Xiao[b], Haixia Zhu[b], Yao Liu[a], Zhaozhe Chen[b], Defeng Xu[b], Weichang Zhou[a], Zhihui Chen[b], Shan Liang[a,*], Hui Tong[c], Xueyi Guo[c] and Jun He[b]**

[a]Department of Physics, Hunan Normal University, Changsha 410081, China

[b]Hunan Key Laboratory of Nanophotonics and Devices, School of physics, Central South University, 932 South Lushan Road, Changsha, Hunan 410083, China.

[c]School of Metallurgy and Environment, Central South University, Changsha 410083, China.

[*]Corresponding author: Liangshan@hunnu.edu.cn (Shan Liang)

Supplementary Figures

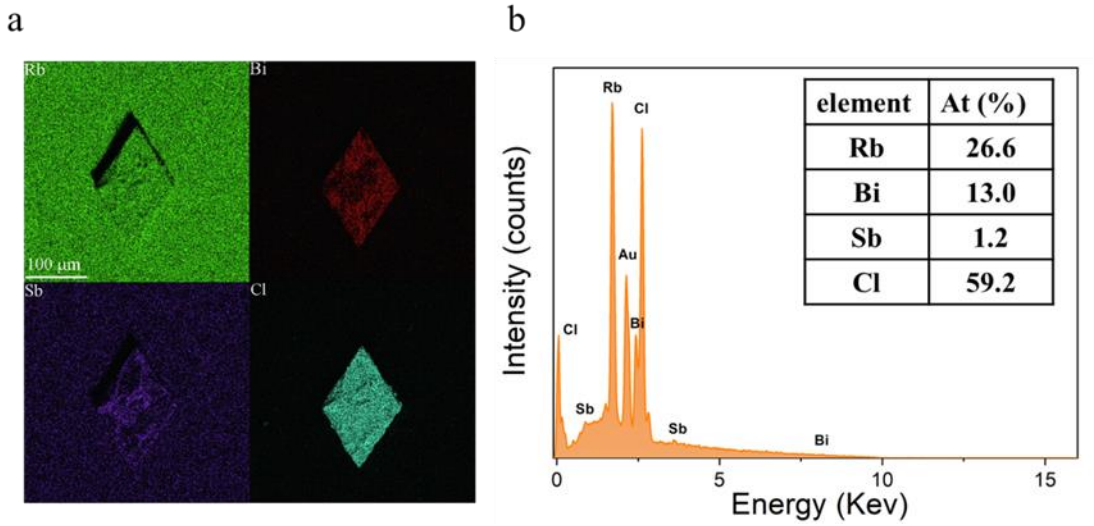

Figure S1. a) Elemental mappings of $Rb_7Bi_3Cl_{16}$: 5% Sb^{3+} nanosheets. b) EDS spectrum of $Rb_7Bi_3Cl_{16}$: 5 % Sb^{3+} crystals.

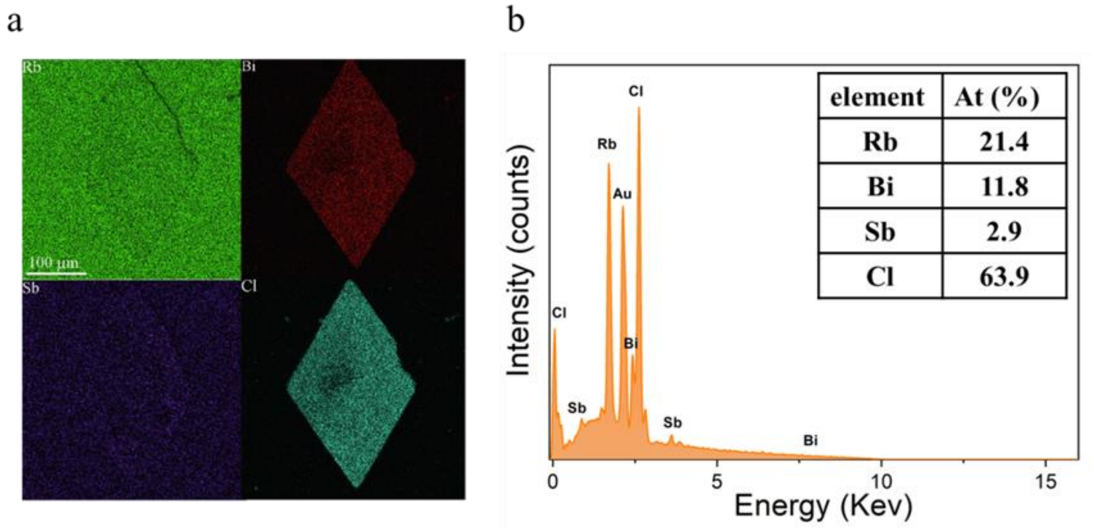

Figure S2. a) Elemental mappings of $Rb_7Bi_3Cl_{16}$:15% Sb^{3+} nanosheets. b) EDS spectrum of $Rb_7Bi_3Cl_{16}$: 15 % Sb^{3+} crystals.

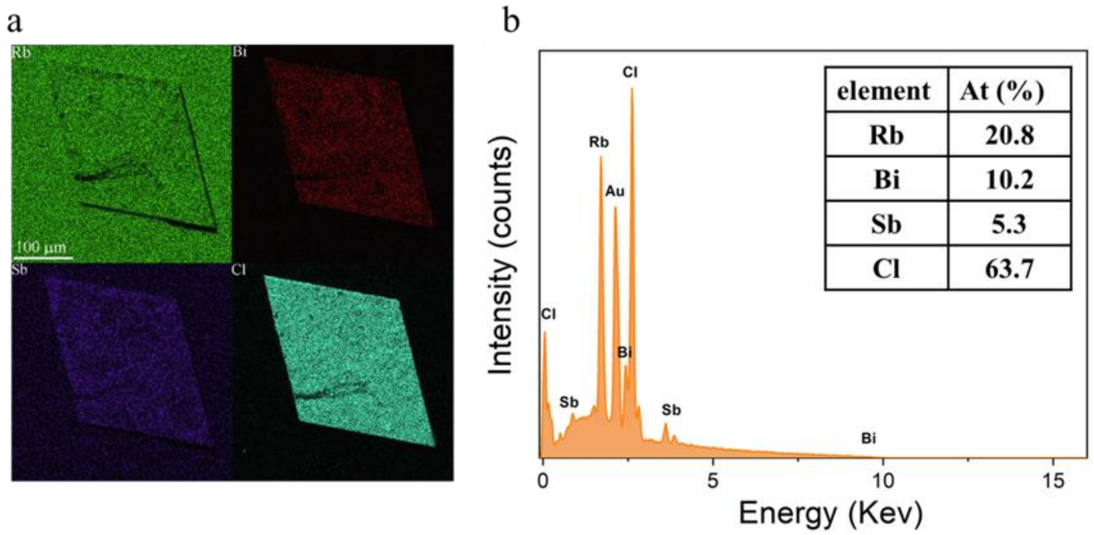

Figure S3. a) Elemental mappings of $Rb_7Bi_3Cl_{16}$: 30% Sb^{3+} nanosheets. b) EDS spectrum of $Rb_7Bi_3Cl_{16}$: 30 % Sb^{3+} crystals.

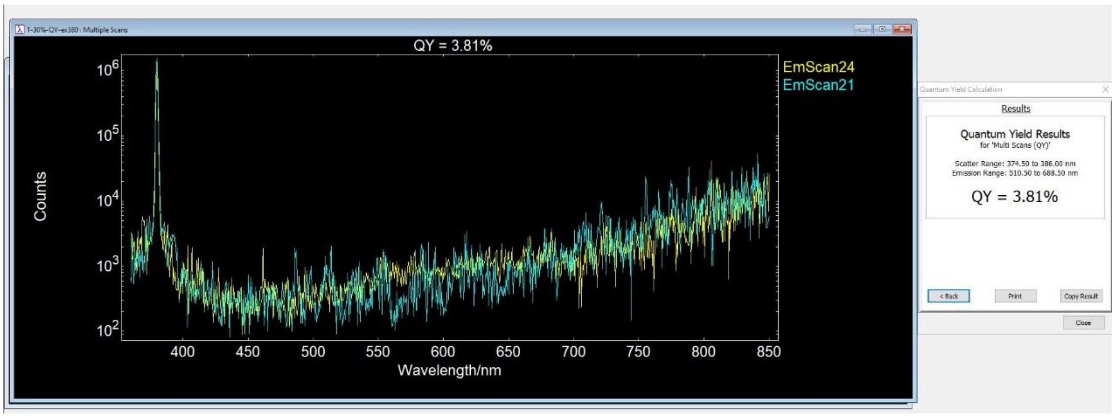

Figure S4. The quantum yield of $Rb_7Bi_3Cl_{16}$:5% Sb^{3+} under 370 nm excitation at RT.

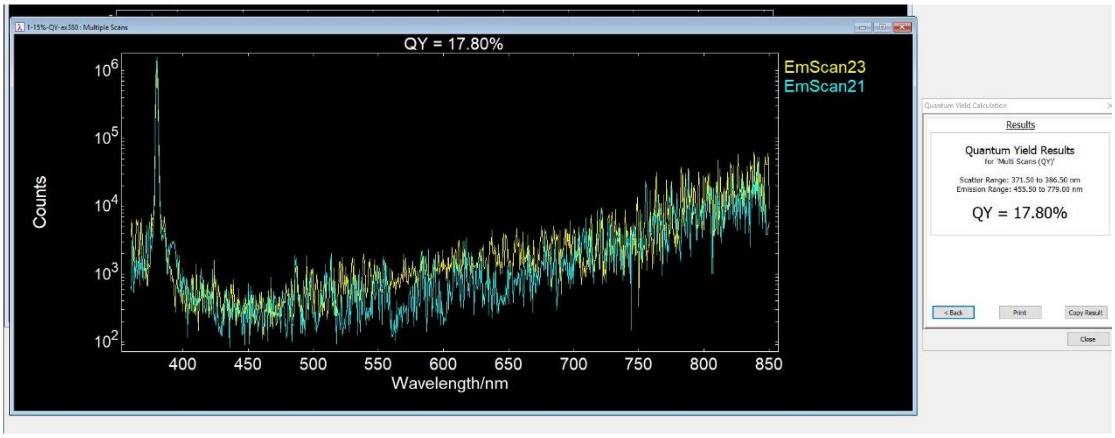

Figure S5. The quantum yield of $Rb_7Bi_3Cl_{16}$:15% Sb^{3+} crystals under 370 nm excitation at RT.

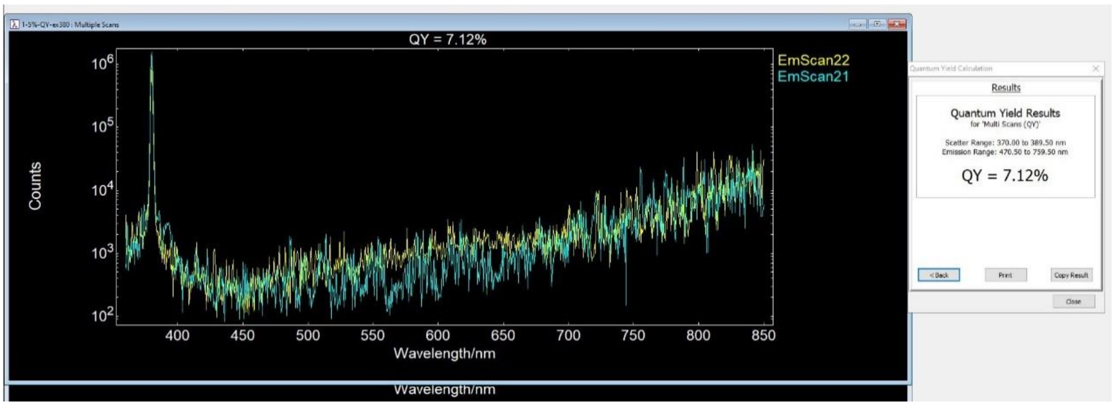

Figure S6. The quantum yield of $Rb_7Bi_3Cl_{16}$:30% Sb^{3+} under 370 nm excitation at RT.

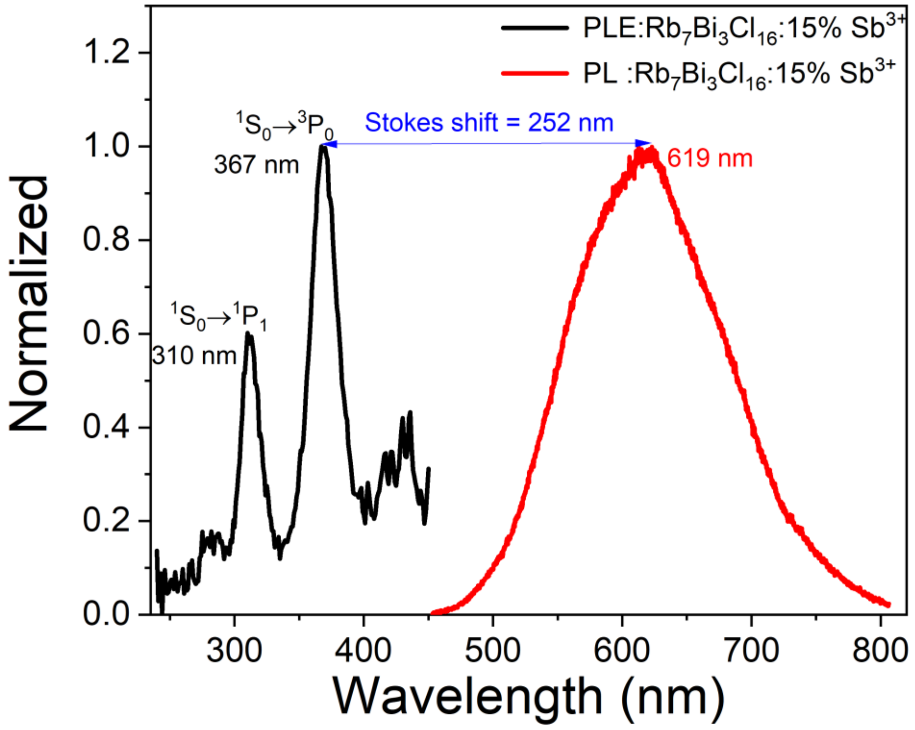

Figure S7. The PLE (black Line) and PL (red line) spectra of $Rb_7Bi_3Cl_{16}$:15% Sb $^{3+}$at RT.

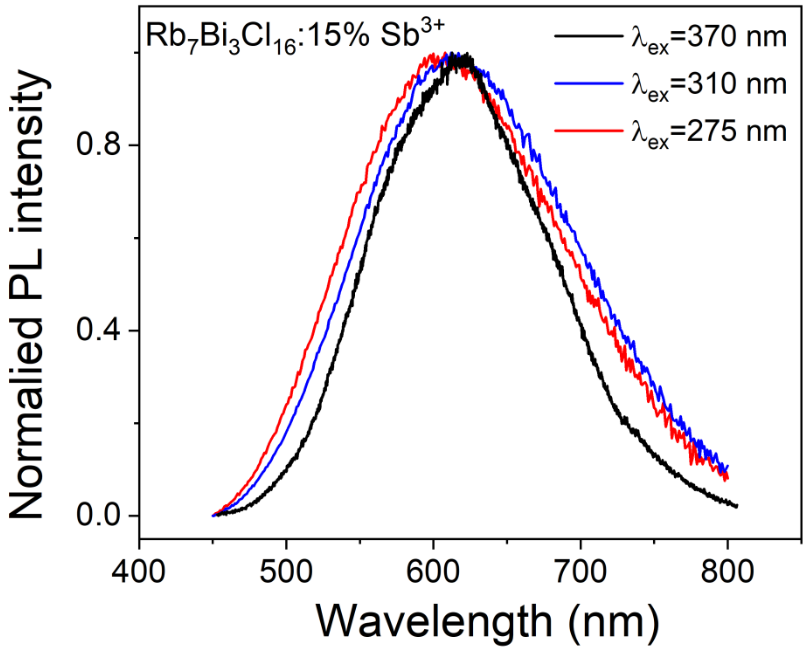

Figure S8. PL spectra (λ_{ex}=275, 310, and 370 nm) of $Rb_7Bi_3Cl_{16}$:15% Sb^{3+} .

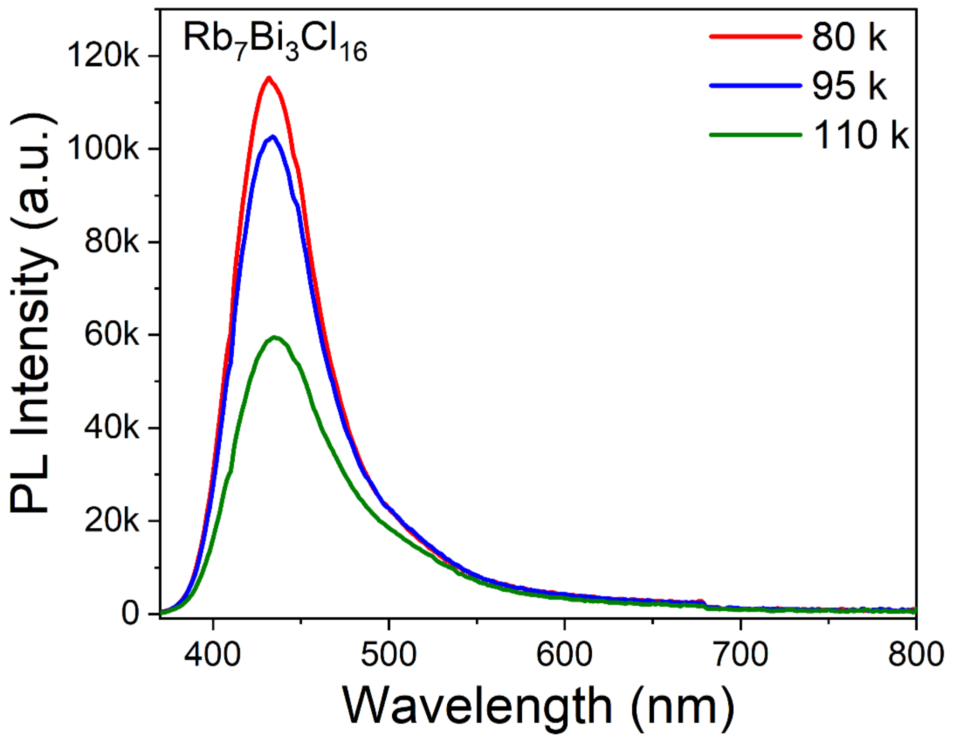

Figure S9. PL spectra of $Rb_7Bi_3Cl_{16}$ under low temperature conditions (80 k, 95 k, and 110 k).

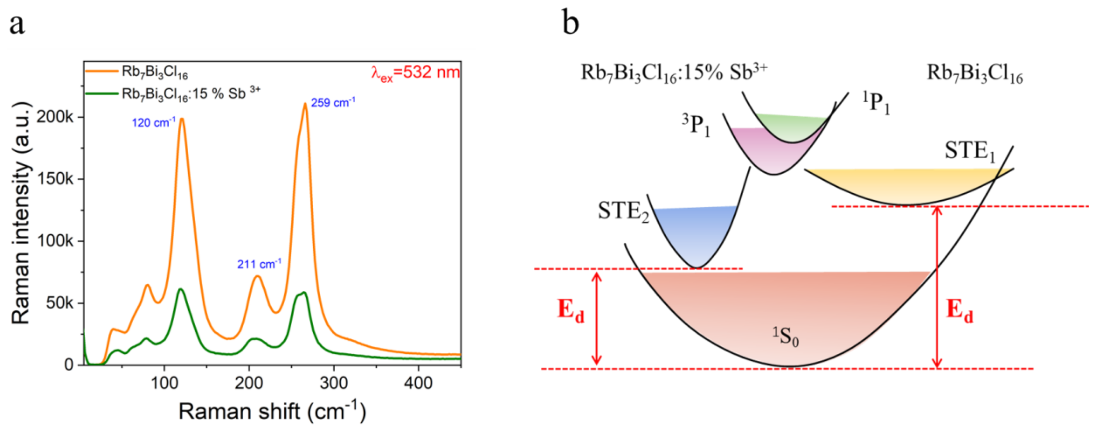

Figure S10. a) Raman spectroscopy of $Rb_7Bi_3Cl_{16}$ and $Rb_7Bi_3Cl_{16}$: 15 % Sb^{3+}, respectively. b) The distortion of $Rb_7Bi_3Cl_{16}$ and $Rb_7Bi_3Cl_{16}$:15%

Sb^{3+}, respectively.

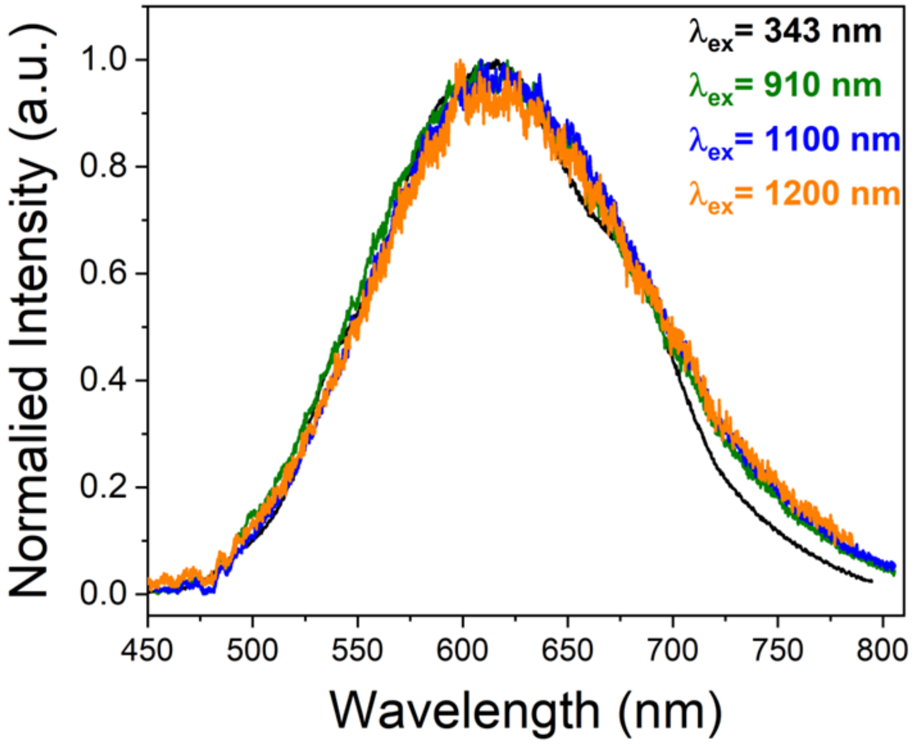

Figure S11. The normalized 1PPL, 2PPL, 3PPL spectrum of $Rb_7Bi_3Cl_{16}$:15 % Sb^{3+}.

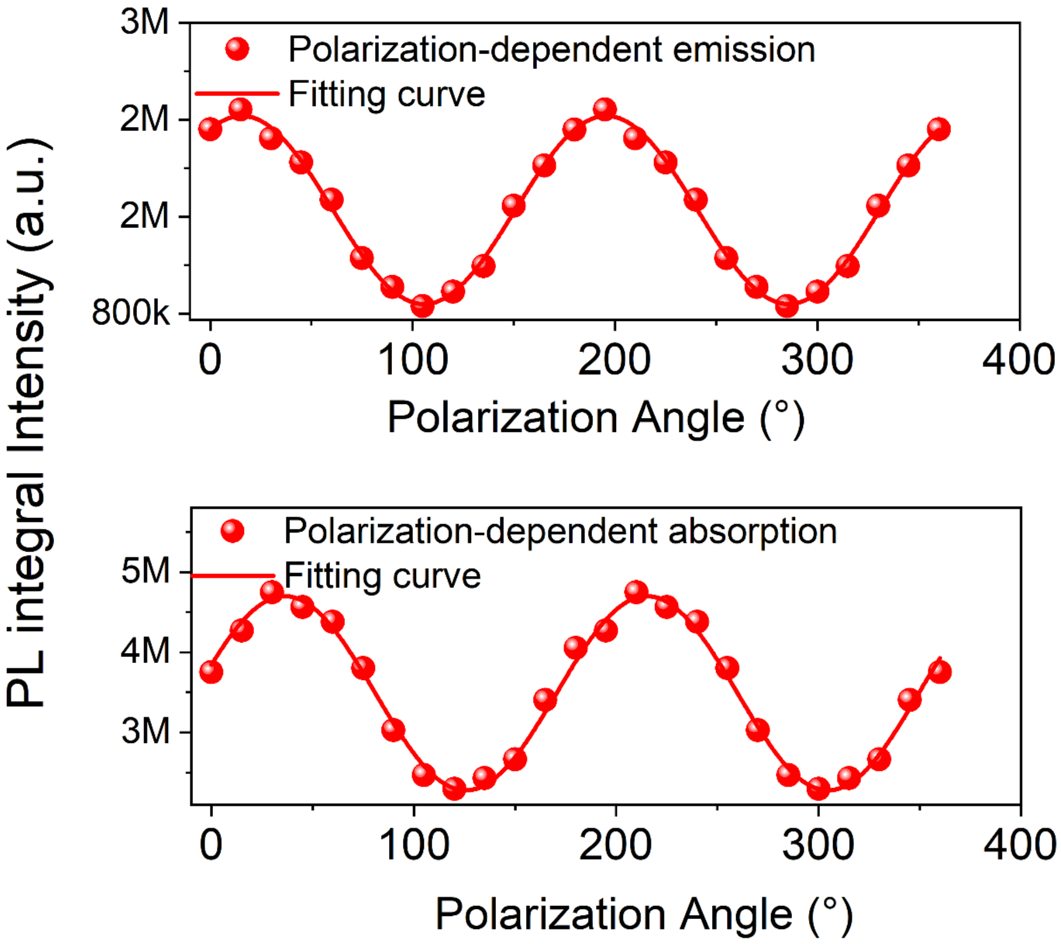

Figure S12. The integrated PL intensity as a function of polarizer for $Rb_7Bi_3Cl_{16}$: Sb^{3+} nanosheets in the polarization-dependent emission (top) and polarization-dependent absorption (bottom).

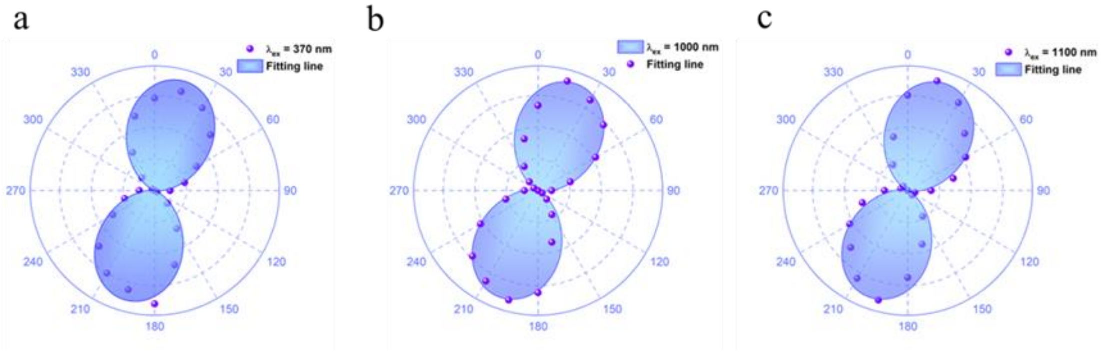

Figure S13. Polar plot of integrated PL intensity of $Rb_7Bi_3Cl_{16}$:15% Sb^{3+} as a function under a) 370 nm, b) 1000 nm, c) 1100 nm excitation wavelengths respectively.
